# Molecular mechanisms of experience-dependent plasticity in visual cortex

**DOI:** 10.1098/rstb.2008.0269

**Published:** 2008-10-31

**Authors:** Daniela Tropea, Audra Van Wart, Mriganka Sur

**Affiliations:** Picower Institute for Learning and Memory and Department of Brain and Cognitive Sciences, Massachusetts Institute of TechnologyCambridge, MA 02139, USA

**Keywords:** ocular dominance plasticity, critical period, synapses, feed-forward regulation, feedback regulation, homeostasis

## Abstract

A remarkable amount of our current knowledge of mechanisms underlying experience-dependent plasticity during cortical development comes from study of the mammalian visual cortex. Recent advances in high-resolution cellular imaging, combined with genetic manipulations in mice, novel fluorescent recombinant probes, and large-scale screens of gene expression, have revealed multiple molecular mechanisms that underlie structural and functional plasticity in visual cortex. We situate these mechanisms in the context of a new conceptual framework of feed-forward and feedback regulation for understanding how neurons of the visual cortex reorganize their connections in response to changes in sensory inputs. Such conceptual advances have important implications for understanding not only normal development but also pathological conditions that afflict the central nervous system.

## 1. Introduction

Plasticity—the ability of the brain to reorganize its connections structurally and functionally in response to changes in sensory experience—is fundamental for the development of neuronal circuitry in central brain structures and for enabling the brain to adapt to its environment. Experience-dependent plasticity embodies the developmental history of the organism and matches neuronal circuits to the nature of inputs to enable appropriate information processing. Importantly, this experience-driven synaptic organization serves as a scaffold for subsequent reorganization underlying learning and memory. Indeed, many of the mechanisms that are involved in developmental plasticity are the forebears of later mechanisms of learning and memory during adulthood in various brain regions. Furthermore, understanding the mechanisms involved in the development and plasticity of connections is important not only for understanding the formation of neural circuitry but also for specifying possible deviations from a common developmental plan, and hence the aetiology of developmental brain disorders.

The visual cortex has long been a proving ground for the study of experience-dependent plasticity because visual experience can be easily manipulated and the consequences of manipulations can be readily measured at the anatomical, physiological and molecular levels. Although the maturation of visual system circuitry starts before the onset of vision, and the targeting of thalamocortical connections occurs at very early developmental stages (Crowley & Katz 1999; Sur & Leamey 2001; Sur & Rubenstein 2005), proper development of the visual system requires sensory experience. In fact the total absence of sensory input leads to a delay in the maturation of the visual cortex. In animals reared in darkness from birth, cortical neurons display immature properties including reduced orientation and direction tuning, larger receptive field sizes, and lower visual acuity typical of immature neuronal properties observed at the time of eye-opening (Fregnac & Imbert 1978; Timney *et al*. 1978; Fagiolini *et al*. 1994). Total lack of visual experience also affects the fine structure of visual cortex neurons, measured as alterations in the size and density of dendritic spines, the post-synaptic elements for the majority of glutamatergic connections (Wallace & Bear 2004). Normal developmental processes seem to be restored once the animals are exposed to light, thus allowing the recovery of neuronal response properties, such as orientation selectivity (Buisseret *et al*. 1982).

In this review we will discuss recent findings regarding the cellular and molecular mechanisms underlying activity-induced changes in visual cortical function. These have been studied predominantly in rodents, owing to the simplicity of the rodent visual system and the relative ease of genetic manipulation. Mice genetically modified to over- or under-express genes of interest have elucidated roles for many key molecules in plastic processes, and continue to serve as important tools for investigating target molecules in plasticity. *In vivo* visualization of the physiology and structural dynamics of synapses and specific cell classes has been made possible by the creation of mice expressing fluorescent reporters of activity, the combination of high-resolution imaging techniques with fluorescent probes of activity, and the introduction of recombinant fluorescent probes for specific molecules. Furthermore, microarray screens have enabled the identification of genetic signatures of development stages, and of novel activity-regulated genes and pathways which potentially mediate plasticity.

## 2. Critical period for ocular dominance plasticity in visual cortex

A classic form of plasticity used as a model for understanding how activity shapes brain circuitry is ocular dominance plasticity: the rapid changes in visual cortex circuitry which result from unbalanced inputs from the two eyes. Hubel & Wiesel (1963) first demonstrated that thalamocortical inputs from the two eyes segregate in primary visual cortex (V1) of cats to form ocular dominance columns. Reducing or blocking input from one eye during development leads to a loss of physiological responses to that eye, and alteration in the pattern of segregation of eye-specific inputs in V1 (Hubel & Wiesel 1965; Stryker & Harris 1986). Interestingly, ocular dominance plasticity is markedly pronounced during a specific developmental time window termed the ‘critical period,’ a finding that has also been confirmed in ferrets (Issa *et al*. 1999) and monkeys (Horton & Hocking 1997). Yet in recent years the exact definition, and even the existence of this period at all in rodents, has been called into question. This controversy is due to the finding that plastic changes in rodents are possible outside of the classically defined critical period, leading to a consensus view of the critical period as a particularly sensitive phase of development during which even brief alterations in visual experience induce significant cortical plasticity (particularly changes in neuronal structure and connectivity). This is made possible by a number of factors that themselves are influenced by activity and experiential history (reviewed by Hooks & Chen (2007) and Morishita & Hensch (2008)). Nonetheless, prolonged periods of altered sensory experience or primed experience can also lead to synaptic modifications beyond the critical period (Rittenhouse *et al*. 1999; Sawtell *et al*. 2003; He *et al*. 2006; Hofer *et al*. 2006). These changes in adults mediate not only the strength of eye-specific drive, but also the response properties of the non-deprived eye (Prusky *et al*. 2006).

**Figure 1:**
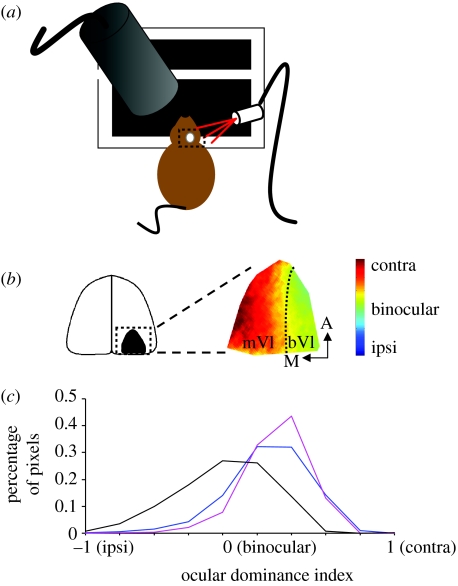
Testing the role of candidate molecules in ocular dominance plasticity using optical imaging of intrinsic signals. Similar measurements of ocular dominance shifts have been made using a number of physiological techniques, such as single unit recordings, visually evoked potentials (VEP) and optical imaging of intrinsic signals (OI). These techniques have been used to evaluate the relative activity evoked by each eye, but while single unit recordings measure spike-related events, VEP and OI also measure synaptic events. Optical imaging provides a particularly rapid and effective measurement of population responses from an expanse of cortex, and can be carried out repeatedly in the same cortex and with minimal invasiveness. (*a*) During intrinsic signal imaging, mice are placed in a stereotaxic apparatus in front of a monitor displaying a periodic drifting bar, and metabolically related changes in light reflectance (630 nm) are captured through the thinned skull with a charge-coupled device camera. Contralateral and ipsilateral eye responses are determined per pixel using Fourier analysis to isolate the component of the response at the stimulus frequency, and are used to define the monocular (mV1) and binocular (bV1) regions of V1. (*b*) An ocular dominance index (ODI) is calculated as the difference between the contralateral eye response and ipsilateral eye response, divided by the summed response, indicating a contralateral (+1) or ipsilateral (−1) bias. (*c*) Under normal conditions, the cortex is more strongly activated by stimulation of the contralateral eye, as indicated by the histogram of pixel ODI values (blue line). Deprivation of this eye during the critical period shifts the cortical activation towards the open, ipsilateral eye (black line). The influence of specific molecules can be evaluated by their effect on this ocular dominance shift. For example, treatment with (1–3) insulin-like growth factor 1 (IGF1; Tropea *et al*. 2006) concurrent with deprivation prevents this shift (pink line), confirming a key modulatory role of IGF1 in ocular dominance plasticity.

Although V1 of rodents does not contain ocular dominance columns, it does contain a discrete binocular segment that has been used extensively to characterize structural and functional rearrangement of cortical circuitry (figure 1). Similar to higher mammals, monocular deprivation (MD) or closure of one eye for as little as 1–2 days during the critical period shifts the physiological responsiveness of neurons in the binocular zone of V1 towards the open eye (Gordon & Stryker 1996). This is at first due to a reversible weakening of deprived-eye connections and reorganization of intracortical connections in the superficial layers (Trachtenberg *et al*. 2000; Trachtenberg & Stryker 2001), and later to a strengthening of non-deprived-eye representations in cortex, accompanied by anatomical reorganization of thalamacortical afferents (Shatz & Stryker 1978; Antonini & Stryker 1993; Antonini *et al*. 1999; Frenkel & Bear 2004). Major progress has been made in recent years towards understanding the cellular and molecular mechanisms that guide these activity-dependent changes.

## 3. Binocular competition, and feed-forward and feedback mechanisms of plasticity

A defining idea of ocular dominance plasticity is that inputs from the two eyes compete within cortex for ‘synaptic space’ or cortical territory (Hubel & Wiesel 1970): in this context, the effects of MD have been mostly studied in the binocular region of V1, where the loss of deprived-eye inputs seems to be balanced by gain of non-deprived-eye inputs. Despite decades of research, however, the mechanism behind binocular competition has remained elusive: what do inputs from the two eyes compete for? Previous studies have implicated activity-dependent uptake of neurotrophins as the mediator of binocular competition (Maffei *et al*. 1992; Cabelli *et al*. 1995), but subsequent experiments have shown that neurotrophins actually have cell-specific effects, such as regulation of inhibitory circuitry, which may provide an alternative explanation of their importance for ocular dominance plasticity (see below). Recent evidence indicates that binocular competition may actually be the consequence of separable processes mediating loss of deprived-eye responses and gain of non-deprived-eye responses (e.g. Frenkel & Bear 2004; Kaneko *et al*. 2008). The mechanisms subserving ocular dominance plasticity may thus comprise a combination of feed-forward synapse-specific changes and cell-wide global feedback changes, which together are manifest as binocular competition, and which allow visual experience to shape and sharpen cortical circuitry while maintaining cellular and network equilibrium.

The classic view of ocular dominance plasticity has considered mainly feed-forward, Hebbian modulations of synaptic strength (Katz & Shatz 1996) at deprived-eye or non-deprived-eye synapses, often relating them to two mechanisms of synaptic plasticity which have been well characterized *in vitro*: long-term potentiation (LTP) and long-term depression (LTD; Stent 1973). In this view, correlated or decorrelated firing of the pre- and post-synaptic neuron leads to a respective strengthening or weakening of synapses; in addition, precisely timed relationships between pre- and post-synaptic action potentials may enhance or weaken synapses by spike-timing-dependent plasticity (Froemke & Dan 2002). Indeed, it is possible to induce plasticity of neuronal responses or of receptive field properties in visual cortex by synchronizing visual stimulation and cortical activation (Fregnac *et al*. 1988; Meliza & Dan 2006). These experiments suggest the hypothesis that the ocular dominance shift induced by MD is largely a Hebbian form of plasticity, in particular involving LTD of deprived-eye inputs and LTP of non-deprived-eye inputs (Heynen *et al*. 2003; Frenkel & Bear 2004).

In support of this model, it has recently been demonstrated that cortical responses to a repeated stimulus strengthen over time, a process that, similar to LTP, is dependent on NMDA (*N*-methyl-d-aspartate) receptor activation and AMPA (α-amino-3-hydroxy-5-methyl-4-isoxazolepropionic acid) receptor trafficking (Frenkel *et al*. 2006). Interestingly, this form of learning is not developmentally regulated, as it is present both in juvenile and adult animals (Frenkel *et al*. 2006). Developmental ocular dominance shifts also share features with LTP, such as the requirement of NMDA receptor activation (Sawtell *et al*. 2003). However, it is unclear whether the observed changes in visually evoked responses arise from direct potentiation of thalamocortical responses or as an indirect consequence of other mechanisms, such as adjustments of GABAergic (γ-aminobutyric acid-mediated) circuitry. Stronger evidence exists that LTD-like mechanisms influence depression of deprived-eye responses: MD shares similar signatures as LTD, and LTD can be induced *in vivo* (Heynen *et al*. 2003). Furthermore, the reduction in deprived-eye responses after lid suture is likely due to Hebbian processes, as monocular inactivation with TTX (which prevents decorrelated inputs) blocks this depression (Frenkel & Bear 2004). Interestingly, the ability to evoke LTD in cortical layer IV decreases over development (Jiang *et al*. 2007) as does the ability to depress deprived-eye responses by MD (Sawtell *et al*. 2003).

Despite the strong evidence for feed-forward Hebbian changes in synaptic strength, these mechanisms alone are unlikely to account for the observed ocular dominance shift. First, a total loss of deprived-eye inputs does not occur, as one might expect if only Hebbian rules applied. Second, increases in open-eye drive are not detected until after the weakening of deprived-eye responses (Frenkel & Bear 2004). Finally, binocular neurons seem to preserve their original level of drive when tested a few days after deprivation, and monocular neurons (within the monocular zone, as well as those within the binocular zone) responding solely to the inactive eye actually increase their responsiveness (Desai *et al*. 2002; Mrsic-Flogel *et al*. 2007). Thus, in addition to synapse-specific changes driven by visual activity, there are likely to be cell-wide, global feedback changes that counter the effects of deprivation in order to preserve a neuron's total excitatory drive.

**Figure 2:**
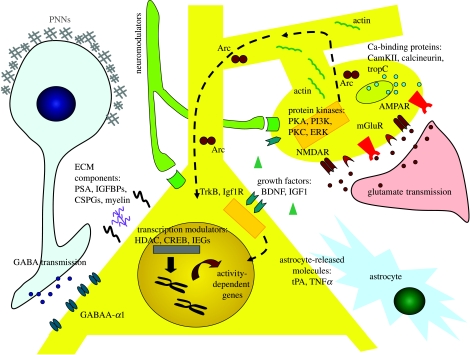
Schematic of key cellular and molecular mechanisms that mediate plasticity in visual cortex. A pyramidal neuron (yellow) receives inputs from a GABAergic neuron (blue, on the left) and from a glutamatergic presynaptic terminal (pink, on the right). The composition and density of GABA and glutamate receptors modulate cortical plasticity, as do the molecules involved in receptor trafficking (Arc). Molecules that detect and bind to post-synaptic calcium, such as cardiac Troponin C, calcineurin and CamKII, are also important for ocular dominance plasticity. Other effectors include MHC (major histocompatibility complex) molecules and growth factors, such as BDNF and IGF1 and neuromodulators (serotonin, acetylcholine and noradrenaline). Changes in calcium influx are followed by signalling cascades that include several protein kinases (such as ERK, PKA and CamKII), and terminate in activation of CREB-mediated transcription. This transcription is further controlled by chromatin-remodelling enzymes. The functional synaptic modifications are coupled with structural rearrangement of dendrites and spines, which most likely is mediated by actin remodelling. At the extracellular level, myelin-related receptors (NogoR) and components of the extracellular matrix (chondroitin sulphate proteoglycans, polysialic acid, insulin-like growth factor-binding protein and tissue plasminogen activator) regulate the capacity for structural plasticity and/or the access of molecular effectors to the cell soma. Some also form nets around inhibitory parvalbuminergic neurons (perineuronal nets, PNNs), which appear to restrict plasticity. Serotoninergic, cholinergic and noradrenergic afferents also modulate visual plasticity. Finally, glial cells (astrocytes) contribute to cortical plasticity by modulating glutamatergic transmission and producing plasticity-related molecules such as IGFBPs, tPA and TNFα. Abbreviations: PNNs, peri-neuronal nets; PSA, polysialic acid; ECM, extracellular matrix; IGFBPs, insulin-like growth factor-1-binding proteins; CSPGs, chondroitin-sulphate proteoglycans; HDAC, histone deacetylases; IEGs, immediate early genes; CREB, cAMP-responsive element-binding protein; tPA, tissue-type plasminogen activator; TNFα, tumour necrosis factor alpha; PKA, protein kinase A; PKC, protein kinase C; PI3K, phosphatidylinositol 3-kinase; ERK, extracellular signal-regulated kinase; tropC, cardiac troponin C; CamKII, calcium/calmodulin-dependent protein kinase II; BDNF, brain-derived neurotrophic factor; Igf1R, insulin-like growth factor 1 receptor; TrkB, tyrosine receptor kinase B.

In this view, feed-forward synapse-specific mechanisms reduce synaptic efficacy at deprived-eye synapses, whereas feedback cell-wide mechanisms upregulate efficacy at other synapses, importantly including those from the non-deprived eye. Together, the effect would be manifest as binocular competition. Indeed, balanced levels of excitation and inhibition are critical not only for enabling plasticity but also for allowing neurons to generate specific response properties or carry out complex synaptic integration (Hensch & Fagiolini 2005; Marino *et al*. 2005). (An alternative proposal for explaining activity-dependent plasticity, known as the BCM rule, also has considerable explanatory power: Bienenstock *et al*. 1982.) The following sections will highlight our current understanding of the very large number of feed-forward and feedback mechanisms by which changes in activity lead to synaptic and network plasticity in visual cortex (figure 2, table 1).

## 4. Mechanisms of feed-forward plasticity

### (a) Glutamatergic receptors

Excitatory transmission is mediated by glutamate-gated AMPA and NMDA receptors, whose number and subunit composition regulate membrane depolarization and intracellular calcium levels, and by mGluR (metabotropic glutamate) receptors, which regulate downstream signalling events. Evidence exists that each of these receptor types may promote plasticity in visual cortex. Calcium influx through NMDA receptors is determined by their subunit composition (NR1 and either NR2A or NR2B subunits), and repetitive activation leads to increased insertion of synaptic AMPA receptors, leading to LTP. The direct dependence of OD plasticity on NR1 subunits has been demonstrated using conditional NR1-knockout mice (Sawtell *et al*. 2003). Visual deprivation also influences the NR2 subunit composition of NMDA receptors, which normally transitions from low to high NR2A/NR2B ratios during post-natal development. Dark rearing or lid suture reduces this ratio, and this change can be reversed upon re-exposure to light (Quinlan *et al*. 1999; Tongiorgi *et al*. 2003; Chen & Bear 2006). This activity-dependent change in subunit composition has also been shown in adult animals, where dark rearing prior to MD can decrease the NR2A/NR2B ratio and promote ocular dominance plasticity, potentially by influencing the threshold for LTP (He *et al*. 2006). Interestingly, NR2B over-expressing animals are not more susceptible to plasticity (Philpot *et al*. 2001), possibly because modulating NR2B transcript did not affect the NR2A/2B ratio in this study. However, mice lacking NR2A subunits had a reduced sensitivity to MD, which could be restored by increasing inhibition with diazepam (Fagiolini *et al*. 2003). These findings suggest that developmental changes in NMDA-mediated excitatory currents can regulate the capacity for experience-dependent plasticity.

AMPA receptors in the brain are primarily composed of GluR2 and either GluR1 or GluR3 subunits. Synaptic strength, including LTP, is significantly determined by AMPA receptor number and calcium permeability (which is also determined by subunit composition; see for review Citri & Malenka 2008). Several studies have demonstrated that subunits of AMPA receptors are preferentially inserted at synapses that undergo LTP and are removed from synapses that undergo LTD (Malinow & Malenka 2002), a process that may occur in visual cortex as well (Heynen *et al*. 2003). However, it is of note that AMPA receptor endocytosis is not required for LTD in all cortical layers. Rather, endocannabinoid signalling to the presynaptic terminal is necessary and sufficient to induce LTD in layer 2/3 of visual cortex (Crozier *et al*. 2007), and blocking layer 2/3 cannabinoid receptors *in vivo* during MD prevents the shift in ocular dominance (Liu *et al*. 2008).

There is also direct evidence that metabotropic glutamate receptors are involved in visual cortex plasticity (Daw *et al*. 1999), with distinct roles depending on the receptor subtype and cortical layer (Wang & Daw 2003; Rao & Daw 2004). For example, reducing mGluR5 receptor levels by 50 per cent in transgenic mice shifts the ocular dominance value towards the non-deprived eye with respect to control mice (Dolen & Bear 2008), suggesting a feed-forward role for the receptor in the reinforcement of plastic rearrangements in the binocular visual cortex after MD.

### (b) Calcium signalling and downstream molecules

Calcium influx induced by synaptic depolarization activates a number of intracellular signalling cascades, which can modify diverse cellular processes in a calcium-dependent manner. Experiments using transgenic mice and/or pharmacological manipulation have identified three signalling kinases that can modulate synaptic strength and are critical for inducing ocular dominance shifts: extracellular signal-regulated kinase 1,2 (ERK, also called p42/44 mitogen-activated protein kinase), protein kinase A (PKA), and calcium/calmodulin-dependent protein kinase II alpha (CaMKIIα; Di Cristo *et al*. 2001; Taha *et al*. 2002; Berardi *et al*. 2003; Taha & Stryker 2005). These kinases may rapidly promote ocular dominance plasticity by directly phosphorylating plasticity-regulating molecules at the synapse (such as glutamate or GABA receptors), thereby modulating synaptic strength, or they may signal to the nucleus to mediate changes in gene transcription.

The intracellular mechanisms mediated by kinase signalling can lead to the activation of cAMP-responsive element-binding protein (CREB), which in turn controls CRE-mediated gene expression of a host of synaptic signalling molecules (Cancedda *et al*. 2003; but see also; Suzuki *et al*. 2004). Visual manipulation (MD) induces activation of CREB (Pham *et al*. 1999); a direct requirement for CREB in ocular dominance plasticity was shown in ferret using viral-mediated expression of a dominant-negative form of CREB (Mower *et al*. 2002). Additional *in vivo* work combining expression of a CRE-driven LacZ reporter with kinase-specific pharmacological blockade showed that while PKA and ERK inhibition affected CRE-mediated gene expression, the effects of PKA were dependent on ERK phosphorylation (Cancedda *et al*. 2003). These results point to ERK as a molecular sensor of visually driven activity. Interestingly, while ERK activation and CRE-gene expression appear to be strongly correlated, it has been shown that ERK activation and phosphorylated CREB do not always overlap (Suzuki *et al*. 2004), suggesting that other co-activators of CREB are important transducers of synaptic activity. As with many other molecules that mediate changes in plasticity, CREB levels also decrease with age (Pham *et al*. 1999). A related finding of particular interest is the finding that activation of CREB is mediated by visual stimulation in young but not adult rats (Putignano *et al*. 2007), demonstrating that different intracellular pathways contribute to cortical plasticity at different ages. Interestingly, several of these signalling molecules have been shown to be involved in activity-dependent plasticity in other cortical areas as well (for a review, see Fox 2002).

These pathways may also converge to mediate structural rearrangements induced by MD. For example, *in vitro* studies have shown that PKA localizes to dendritic spines and is involved in actin reorganization upon NMDAR activation (Gomez *et al*. 2002; Hsieh-Wilson *et al*. 2003), while ERK controls neurite outgrowth (Chierzi *et al*. 2005) and is required for BDNF-dependent increases in spine density (Alonso *et al*. 2004).

Additional classes of molecules are also likely to be important for calcium-dependent cellular processes that may mediate brain plasticity. For example, one additional link between calcium signalling and cytoskeletal dynamics comes from a recent microarray screen, which has found that the calcium sensor cardiac troponin C (part of a complex that mediates calcium-dependent actin–myosin interaction) is elevated in visual cortex during the critical period, and is regulated by visual activity (Lyckman *et al*. 2008). Additionally, calcineurin, a calcium/calmodulin-activated phosphatase, has proven to be an effective negative regulator of ocular dominance plasticity: calcineurin overexpression reversibly prevents an ocular dominance shift during the critical period in mouse (Yang *et al*. 2005). Thus, a balance of calcium-dependent kinase and phosphatase activity appears to be important for deprivation-induced synaptic reorganization.

### (c) GABAergic inhibition and BDNF signalling

GABA-mediated inhibition regulates cortical plasticity on multiple fronts. Maturation of cortical inhibition is strongly involved in the timing of the critical period for ocular dominance plasticity (Hensch 2005) as well as ocular dominance column development in the cat (Hensch & Stryker 2004). There is now considerable evidence that a minimal level of inhibition is necessary for the initiation of ocular dominance plasticity, and that factors that influence the development and extent of GABA transmission (such as BDNF, benzodiazepines, PSA-NCAM and fluoxetine) can control the plastic properties of visual cortical circuitry (Hensch & Fagiolini 2005; Jiang *et al*. 2005; Di Cristo *et al*. 2007; Maya Vetencourt *et al*. 2008). Moreover, BDNF infusion during MD is able to re-induce plasticity in adult rats, possibly through a decrease in GABAergic transmission (Maya Vetencourt *et al*. 2008).

These recent pharmacological studies have focused much attention towards a specific subset of GABAergic neurons, the parvalbumin-positive cells (which include fast-spiking basket cells), for their role in visual plasticity (Fagiolini *et al*. 2004; Maffei *et al*. 2006; Tropea *et al*. 2006). For example, maturation of these cells is regulated by BDNF (Huang *et al*. 1999), and the benzodiazepine-sensitive GABA_A_-α1 subunits are localized on receptors that specifically receive parvalbumin-positive afferents (Klausberger *et al*. 2002). Mice lacking these receptors have more sustained GABA currents, an effect similar to the administration of benzodiazepines (Ponomarev *et al*. 2006). In addition, fast-spiking basket cells have been shown to mediate potentiation of inhibition in visual cortex *in vitro*, suggesting an important feed-forward mechanism that contributes to the rapid deprived-eye depression following MD (Maffei *et al*. 2006).

There is also structural evidence for a role of GABA-ergic transmission in synaptic development and plasticity. For example, the reduction in spine density normally evoked by MD is not observed in GAD65 knockout mice, which seem to be unaffected by MD (Mataga *et al*. 2004). In addition, mice lacking the GABA_(A)_-α1 subunit display an increased density of filopodia and a decreased density of stable mushroom spines between two and three weeks after birth (Heinen *et al*. 2003), resembling the morphological features typical of immature circuitry. The differential capacity for changes within the excitatory versus inhibitory network over development may contribute to the differences between juvenile and adult plasticity. For example, the reorganization of dendritic arborization in adult mice is restricted to GABAergic inter-neurons, while glutamatergic cells lose this ability (Lee *et al*. 2006). Furthermore, conditions that alter GABAergic transmission in the adult, such as an enriched environment (Sale *et al*. 2007) and fluoxetine administration (Maya Vetencourt *et al*. 2008), are able to re-induce ocular dominance plasticity in adult rodents.

### (d) Structural plasticity: spine dynamics and the extracellular environment

A critical locus for physiological and anatomical changes in glutamatergic transmission is at the level of dendritic spines, the structures that receive the majority of excitatory inputs in the CNS. Owing to advances in multi-photon microscopy and the use of molecular technologies for labelling cells *in vivo*, considerable insights have been gained into the link between synaptic activity and spine morphology and dynamics in the brain. In rodents, sensory experience affects both structure and dynamics of dendritic spines (Lendvai *et al*. 2000; Zito & Svoboda 2002). In visual cortex, spine density is reduced in the binocular area after brief MD (Mataga *et al*. 2004), suggesting a correlation between spine loss and rapid reduction in deprived-eye drive. Likewise, *in vivo* structural imaging of green fluorescent protein-labelled neurons in ferret V1 combined with functional delineation of ocular dominance regions demonstrates that functional changes after deprivation are accompanied by a significant, but reversible, loss of dendritic spines (Yu *et al*. 2005). Spine dynamics have been shown to decrease by AMPA and/or NMDA application in visual cortical slices, suggesting spines are stabilized by synaptic activation (Oray *et al*. 2006). Importantly, spine loss and morphological changes have also been observed in dark-reared animals (Wallace & Bear 2004) and spine motility is altered in an age-specific manner in binocularly deprived animals (Majewska & Sur 2003), suggesting that competition between the two eyes is not fundamental for the reorganization of dendritic spines.

The quantity and dynamics of dendritic spines are heavily influenced by molecules that act on the extracellular matrix, such as chondroitinase ABC and tissue plasminogen activator (tPA). The expression of extracellular perineuronal nets (PNNs) in visual cortex matches the development of the critical period, is delayed by dark rearing and is restricted mostly to GABA-expressing neurons (Guimaraes *et al*. 1990; Hartig *et al*. 1992). Using chondroitinase ABC to selectively degrade chondroitin sulphate proteoglycans in the PNNs, it is possible to induce an ocular dominance shift in adult animals (Pizzorusso *et al*. 2002), suggesting that in adults, PNNs normally prevent the reorganization of the circuitry that would occur during ocular dominance plasticity.

A similar role has been proposed for tPA, a serine protease that is present in neurons and is released in an activity-dependent manner (Mataga *et al*. 2002). Degradation of the extracellular matrix with the tPA/plasminogen proteolytic cascade prevents the loss of superficial spines normally induced by 4 days of MD (Mataga *et al*. 2004)—an effect that is not observed in tPA knockout mice. tPA application also mimics the enhancement of spine dynamics seen after brief (2 days) MD (Oray *et al*. 2004). Importantly, mice deficient for tPA fail to produce any ocular dominance shift. These data point to an important feed-forward role for tPA in ‘freeing up’ the extracellular matrix to promote the structural reorganization of connections during deprivation.

Another extracellular factor that regulates the capacity for ocular dominance plasticity is myelin from the surrounding oligodendrocytes, particularly via its interaction with the Nogo receptor (McGee *et al*. 2005). The critical period window for ocular dominance plasticity is substantially extended in Nogo receptor-null mice, despite the normal development of other factors that regulate plasticity, such as tPA levels and GABAergic transmission. Interestingly, cortical myelination does not appear to be regulated by visual experience, as dark-rearing wild-type mice does not affect the expression level of myelin-related proteins. Furthermore while visual deprivation alters transcription of a number of plasticity-related molecules, developmental increases in myelin-associated genes remain unchanged (Lyckman *et al*. 2008).

### (e) Effects of neuromodulatory systems on cortical plasticity

Several studies have aimed to uncover the contribution of neuromodulators to cortical plasticity, particularly in relation to feed-forward mechanisms. It has been known for over 30 years that agonists of adrenergic and cholinergic systems facilitate the onset of ocular dominance plasticity (Kasamatsu & Pettigrew 1976; Bear & Singer 1986; for review see Gu 2003), and later an analogous function was described for the serotoninergic system (Gu & Singer 1995). These systems are also important for the basic function of visual cortex, since lesions in the brain regions that generate the fibres (basal forebrain for cholinergic system and locus coeruleus for noradrenergic afferents) alter the ocular dominance properties and the orientation selectivity of cortical neurons (Siciliano *et al*. 1999), especially when made early in development. Interestingly, administration of the selective serotonin re-uptake inhibitor fluoxetine has been shown to restore ocular dominance plasticity to adults, possibly due to a correlative reduction in inhibition (Maya Vetencourt *et al*. 2008).

As with many other molecules involved in cortical plasticity, the distribution of different receptors and fibres is developmentally regulated (Foote & Morrison 1984: noradrenergic fibres) and dependent on cortical input (Prusky *et al*. 1988: cholinergic receptors). A most interesting observation concerning the spatio-temporal distribution of neuromodulators and their receptors is that the expression of serotonin receptors in kitten visual cortex is organized in patches and is complementary to the cytochrome oxidase staining for ocular dominance columns (Kojic *et al*. 2000). Neuromodulators also control the morphological reorganization of the circuitry, since noradrenaline and serotonin application modulates the number of synapses in an age-dependent manner (Matsukawa *et al*. 2003). This may be due to their ability to modulate thresholds for LTP/LTD induction. *In vitro* application of serotonin facilitates both LTP and LTD induction in slices derived from adult cats (Kojic *et al*. 1997; but see also Edagawa *et al*. 2001) and concurrent stimulation and application of carbachol or noradrenaline induces LTD in visual cortical slices (Kirkwood *et al*. 1999).

A plausible explanation for the effects of neuromodulators on visual cortex plasticity is their ability to uniquely modify the intracellular calcium concentration via second messenger pathways, potentially changing the requirements for LTP/LTD (Kirkwood *et al*. 1999; Kobayashi *et al*. 1999), although for the cholinergic system, it has been shown that different muscarinic receptors activate distinct intracellular pathways (Origlia *et al*. 2006). Thus, the same stimulus may alter plasticity in unique ways depending on the relative contribution of neuromodulatory systems. Further, these systems may also selectively interact with growth factors to affect plastic changes. For example, acetylcholine fibres host the majority of the receptors for the neurotrophin nerve growth factor, and may thereby mediate the effects of this growth factor on ocular dominance plasticity (Maffei *et al*. 1992; Rossi *et al*. 2002).

## 5. Mechanisms of feedback plasticity

The observation that ocular dominance plasticity could not be explained simply with feed-forward mechanisms alone led to the speculation that feedback mechanisms may also exist to regulate plasticity. One of the first ideas invoking a form of feedback plasticity attempted to explain synaptic plasticity in visual cortex according to the BCM theory (Clothiaux *et al*. 1991). This theory (or learning rule) proposes that the response of a system to external manipulations depends on an internal threshold that is not fixed but rather slides as a function of post-synaptic activity. This theory helps explain several findings in mice (Kirkwood *et al*. 1996; Rittenhouse *et al*. 1999; Frenkel & Bear 2004) and in cats, such as the observation that recovery after MD occurs faster when the deprivation is followed by binocular stimulation rather then reverse lid suture, and that readjustment of the threshold takes longer (hours) than Hebbian plasticity (Mitchell *et al*. 1984). However, as described below, recent work suggests that feedback plasticity may actually be a process distinct and separable from feed-forward plasticity.

### (a) Network homeostasis

The absolute drive onto a cortical neuron changes dynamically as feed-forward adjustments are made to synapse number, synaptic weight and circuit organization. Consequently, a number of cell autonomous and non-cell autonomous feedback mechanisms are employed in order to maintain balanced network excitability and preserve effective information transmission, including synaptic homeostasis, changes to intrinsic excitability and regulation of inhibitory drive. Such mechanisms are an integral part of activity-dependent plasticity not only in visual cortex but also throughout the developing nervous system (reviewed in Turrigiano & Nelson 2004). In recent years, a number of diverse signalling molecules have been identified that mediate these feedback responses at particular loci within the cortical circuit, depending on the developmental age, type of visual manipulation and direction of feed-forward plastic changes (towards net excitation or net depression).

### (b) Synaptic homeostasis during ocular dominance plasticity

The best-studied feedback mechanism is synaptic scaling, in which deviation of the cell from a preferred set point for firing rates induces a global scaling up or down of synaptic strengths, without disrupting relative synaptic weights (Turrigiano & Nelson 2004). Interestingly, this scaling is lamina specific, as scaling of miniature excitatory post-synaptic current (mEPSC) amplitudes in layer 4 after activity blockade is restricted to a pre-critical period, while scaling in layer 2/3 is not evident prior to the critical period (Desai *et al*. 2002). Further, while adult brains are typically thought of as less plastic than juveniles, synaptic scaling can be evoked by brief binocular deprivation all the way through adulthood, although this scaling appears to be of a different form (non-multiplicative) and may therefore use mechanisms different from those during the critical period (Goel & Lee 2007).

The exact feedback mechanisms evoked by activity blockade will probably depend on both the duration and strength/quality of deprivation. For example, while two days of monocular inactivation with tetrodotoxin (TTX) leads to a scaling up of layer 2/3 pyramidal mEPSC amplitudes in the affected monocular cortex, the same duration of lid suture instead leads to the scaling of intrinsic excitability, leaving mEPSC strength unmodified (Maffei & Turrigiano 2008). These manipulations also lead to different changes in excitatory/inhibitory balance in the underlying cortical network. Therefore, although both manipulations produce an increase in the spontaneous firing, the mechanisms by which this is achieved are different. Interestingly, deprivation-induced increases in visual responses (via 4–6 days of lid suture or dark rearing) are prevented in mice that lack mechanisms of synaptic scaling (Kaneko *et al*. 2008; Van Wart *et al*. 2008), suggesting that the mechanisms or time scale of feedback events are differentially regulated *in vivo.* Future studies are necessary to understand how these different negative feedback mechanisms are elicited, and how they may converge to stabilize cortical circuitry.

### (c) Molecular mechanisms of synaptic scaling: TNFα and Arc

While relatively little is known about mechanisms governing intrinsic excitability, several activity-dependent molecules have been identified as mediators of synaptic scaling. Positive and negative scaling of mEPSC amplitudes can be mediated by molecules that alter the GluR content at synaptic membranes (Goel *et al*. 2006; Rial Verde *et al*. 2006; Shepherd *et al*. 2006). Malenka and colleagues have shown in hippocampus *in vitro* that after 48 hours of activity blockade, glia increase their production of a cytokine called tumour necrosis factor alpha (TNFα), which in turn leads to increased synaptic GluR1 and scaling of mEPSC amplitudes (Stellwagen *et al*. 2005; Stellwagen & Malenka 2006). TNFα has recently been shown to be critical for feedback plasticity in visual cortex, since lack of TNFα eliminates the enhancement of non-deprived-eye input in deprived binocular cortex that counters the reduction in deprived-eye drive (Kaneko *et al*. 2008). Interestingly, TNFα-mediated scaling is also critical for countering a surprising feed-forward depression that occurs during a brief (4–5 days) binocular deprivation (Van Wart *et al*. 2008). These data highlight a novel role for glia in directly mediating the effects of sensory deprivation in visual cortex.

Interestingly, TNFα was found to positively regulate the membrane expression of β3 integrin, another modulator of synaptic scaling (Cingolani *et al*. 2008), suggesting that these molecules may converge to promote feedback enhancement of responses during activity deprivation. While these molecules are critical for synaptic scaling during activity blockade, additional cell autonomous mechanisms are also necessary, such as reduced CaMKIV activation and transcriptional changes (Ibata *et al*. 2008). Interestingly, one of the substrates of CaMKIV is CREB, a proven modulator of feed-forward plasticity.

A complementary role has been revealed for neuronally expressed Arc (Arg3.1). Arc is an immediate early gene induced by neuronal activity (for review, see Tzingounis & Nicoll 2006). Arc mRNA is transported throughout neurons, and Arc protein can be synthesized in dendrites (Dynes & Steward 2007). During periods of increased activity, Arc interacts directly with dynamin to enhance the rate of endocytosis of AMPARs (Chowdhury *et al*. 2006; Rial Verde *et al*. 2006), thereby reducing total synaptic strength while leaving relative synaptic weights unchanged. Owing to its role in glutamate receptor endocytosis, experiments in which Arc is over-expressed or deleted have demonstrated that an appropriate level of Arc expression is critical for permitting the scaling induced by activity blockade (Rial Verde *et al*. 2006; Shepherd *et al*. 2006). Unlike TNFα, Arc may also influence Hebbian forms of plasticity, but may function explicitly in tandem with feedback mechanisms. Recent studies have used an MD paradigm to begin to understand the complex function of Arc *in vivo*. For example, it has been shown that OD plasticity is prevented in juvenile Arc-null mice, as even 7 days of deprivation are insufficient to elicit either a feed-forward reduction in deprived-eye responses or enhancement of non-deprived-eye responses (McCurry *et al*. 2007).

The role of feedback mechanisms under less extreme modification in synaptic drive will be of great interest. For example, while the initial establishment of orientation selectivity in V1 does not require visual input (Hubel & Wiesel 1963), the selectivity of neurons is enhanced by visual experience (reviewed in Sur & Leamey 2001), a process that requires an increase in response at the preferred orientation of a neuron and a reduction of response at non-preferred orientations. Might feedback mechanisms mediated by Arc and TNFα also be important during this process of selective weakening and strengthening of synapses? Responses from neurons in visual cortex of Arc-null mice suggest that this may be the case, as lack of Arc causes an increase in the percentage of neurons with low orientation selectivity and broader tuning curves, yet normal average firing responses to their preferred orientation (Wang *et al*. 2006). A complementary role for TNFα in shaping visual responses is yet to be examined.

### (d) Feedback regulation of inhibition

In addition to a global scaling-up at glutamatergic synapses, inhibitory circuitry also undergoes feedback modifications complementary to feed-forward changes. For example, in response to a reduction in synaptic drive, interneurons that feed inhibition back onto pyramidal cells see a reduction in strength. This has been shown in cultures (Kilman *et al*. 2002) where two days of activity deprivation increases excitatory transmission and decreases the amplitude of miniature inhibitory post-synaptic currents (mIPSCs), together with a decrease in immunostaining for post-synaptic GABA receptors. Similarly, in slice preparations, there is an increase in the excitatory/inhibitory ratio in layers 2/3 in response to intra-ocular application of TTX (Maffei & Turrigiano 2008). The mechanisms mediating this disinhibition are unclear, but may involve TNFα, which has also been shown to regulate GABA_(A)_ receptor endocytosis (Stellwagen *et al*. 2005). On the flip side, *increased* activity can lead to release of BDNF from pyramidal neurons, and a BDNF-dependent strengthening of excitatory synapses onto GABAergic cells (Rutherford *et al*. 1998; Turrigiano & Nelson 2004). This could lead to an overall reduction in the excitatory/inhibitory balance, particularly when combined with a potential Arc-mediated scaling down of recurrent excitatory connections.

## 6. Molecular pathways and gene systems mediating plasticity

In the past few years, several studies have investigated the molecular mechanisms of visual cortex plasticity using genetic screens, and have opened the door for examination of new families of molecules in plasticity. These studies have analysed the expression patterns of hundreds or thousands of genes using unbiased, state-of-the-art methods for the detection of transcripts, such as differential display (Ossipow *et al*. 2004) and microarray technology (Majdan & Shatz 2006; Tropea *et al*. 2006; Lyckman *et al*. 2008).

Large-scale gene expression studies differ from one another in a number of ways, such as sample selection (the extent of cortex included in samples), the nature of the manipulation, criteria for gene selection and so on. Considering all these factors, together with the intrinsic variability of gene expression analysis (e.g. the limited number of samples), differences across studies are to be expected. Yet there are significant and interesting overlapping findings in the most recent studies of gene expression in visual cortex. Studies that examined gene expression during development (Ossipow *et al*. 2004; Lyckman *et al*. 2008) found an increase in myelin-related genes during the critical period. Analogously, authors who examined changes of gene sets after input modification (Majdan & Shatz 2006; Tropea *et al*. 2006) both identified the MAPK pathway and molecules related to the IGF1 pathway.

In general, all the gene expression studies in visual cortex have examined the broader transcriptional signatures of activity-dependent gene expression with three goals in mind: first, to examine whether the enhanced capacity for plasticity during the critical period is determined by a particular genetic signature, and thus specify the underlying conditions that allow plasticity mechanisms to function; second, to know which genes mediate plasticity, by examining transcriptional changes after visual deprivation; and third, to use the information on novel gene expression patterns to examine whether they translated into functional changes during visual deprivation. These analyses have revealed surprisingly new molecular mechanisms underlying both normal development and visual deprivation-induced plasticity (Ossipow *et al*. 2004; Majdan & Shatz 2006; Tropea *et al*. 2006; Lyckman *et al*. 2008).

Visual deprivation regulates expression of certain signalling molecules (e.g. MAPK signalling) at all ages, but the presence of age-specific gene sets probably shapes the influence of common signalling molecules on brain plasticity (Majdan & Shatz 2006). Further, age-specific gene sets are also governed by the prior experience of the animal. In an interesting analysis of the interplay between ‘nature’ and ‘nurture’, it has been found that many of the genes upregulated during the critical period serve functions that would promote synapse stability (e.g. actin stabilization and myelination), while MD reversed expression of nearly all these ‘critical period genes’ (Lyckman *et al*. 2008). These findings indicate an expectation of electrical activity during the critical period, and hence a propensity towards synapse stabilization during normal rearing, but a reversal towards synaptic rearrangements during visual deprivation, a process that may require an exquisite re-balancing of growth-promoting and growth-restrictive processes.

In order to distinguish between genes driven by different visual manipulations, another screen compared the effects of dark-rearing versus short-term (4 days) and long-term (16 days) MD, compared with critical-period-aged mice (Tropea *et al*. 2006). Dark-rearing from birth led to an increase in the expression of genes guiding synapse formation and synaptic transmission and a reduction in those associated with inhibition, consistent with a delayed maturational state. Fascinatingly, gene sets specifically upregulated by MD were related to growth factor and immune/inflammation system signals, with the latter being particularly enriched after long-term deprivation, indicating a potentially novel feedback role for inflammatory signalling.

In support of this idea, Tropea *et al*. (2006) found that cortical levels of a specific insulin-like growth factor (IGF1)-binding protein, IGFBP5, were upregulated contralateral to the deprived eye, and that deprivation-induced increases in IGFBP5 were critical for the ocular dominance shift. Increasing the IGF1/IGFBP5 ratio through exogenous IGF1 prevented the effects of MD, suggesting that reduced IGF1 signalling is a necessary step for eliciting the plastic changes evoked by MD. Conversely, inflammation/immune signalling pathways such as JAK/STAT (Janus kinase/signal transducer and activator of transcription) and paired-immunoglobulin-like receptor B (PirB)/MHC seem to be important for limiting the extent of ocular dominance plasticity (Syken *et al*. 2006; Van Wart *et al*. 2007).

It will be interesting to see whether the above molecules that limit plasticity during the critical period play a similar role in adult plasticity. For example, earlier experiments in adult cats (Obata *et al*. 1999) found that IGF1 and BDNF are upregulated in the deprived region of visual cortex after a retinal scotoma. However, the same molecules are instead downregulated in juvenile mice in response to a decrease in visual input (Bozzi *et al*. 1995; Tropea *et al*. 2001).

## 7. Conclusions

Ocular dominance plasticity is a powerful model for deciphering the roles of candidate molecules and mechanisms in mediating activity-dependent changes in the cortex during development. Such plasticity invokes a complex, interrelated set of mechanisms, involving a large number of molecules of different classes (table 1, figure 2). Expression of most of these molecules is developmentally regulated (Majdan & Shatz 2006; Lyckman *et al*. 2008) and differentially altered by sensory experience (Majdan & Shatz 2006; Tropea *et al*. 2006). A conceptual framework for understanding the roles of these molecules is to consider their function in the context of feed-forward and feedback mechanisms, which mediate the synapse-specific and global modifications that lead to synaptic, cell and ultimately circuit-level plasticity. Together, these mechanisms translate information from the external world into networks that are adaptively shaped to process that information.

An important goal for the field of cortical plasticity is now to understand how the many molecular mechanisms guiding feed-forward and feedback plasticity are recruited, how they interact and converge to permit and instruct plasticity, and over which time scales they act. Central to this is deciphering how a neuron's preferred ‘set point’ for firing rates is established, and how far or for how long the cell must deviate before feedback mechanisms are initiated. How does a cell sense and compare current excitability with desired excitability? Further, are the same molecular mechanisms involved in adult and developmental plasticity, and does this hold true across different species? The answers to these and related questions will require novel tools and approaches, and will undoubtedly lead to a deeper understanding of how nature and nurture interact to shape the cortex.

## Figures and Tables

**Table 1:** Endogenous molecules recently shown to have a role in visual cortex plasticity. (Recently studied molecules, and the publications describing their role, are listed according to function: extracellular components, transcriptional modulators, calcium-binding proteins, nuclear factors, kinases, growth factors, GABAergic and glutamatergic transmission, neuromodulators and others. ECM, extracellular matrix; E/I, excitatory/inhibitory.)

class of molecules	proposed mechanism	references
*extracellular matrix*		
CSPGs	consolidation of ECM	Pizzorusso *et al*. (2002, 2006)
PSA	maturation of inhibition	DiCristo *et al*. (2007)
IGFBPs	modulation of growth factors	Tropea *et al*. (2006)
TPA	cleavage of ECM molecules/BDNF	Mataga *et al*. (2002, 2004) and Oray *et al*. (2004)
myelin-related receptors	consolidation of circuitry	McGee *et al*. (2005)
*nuclear factors*		
CREB	gene transcription	Pham *et al*. (1999), Mower *et al*. (2002), Cancedda *et al*. (2003) and Suzuki *et al.* (2004)
Arc	activation of cellular signalling/scaling	Tagawa *et al*. (2005), Wang *et al*. (2006) and McCurry *et al*. (2008)
IEGs	activation of cellular signalling	Lachance & Chaudhuri (2004) and Mataga *et al*. (2004)
*transcription modulators*		
HDAC	chromatine rearrangement/gene expression	Putignano *et al*. (2007)
*calcium-binding proteins*		
CamKII	modulator of calcium-dependent signalling	Taha *et al*. (2002)
calcineurin	modulator of calcium-dependent signalling	Yang *et al*. (2005)
troponin C	not known	Lyckman *et al*. (2008)
*kinases*		
PKA	activation of cellular signalling	Beaver *et al*. (2001)
CamkII	modulator of calcium-dependent signalling	Taha *et al*. (2002)
ERK	induction of LTP	DiCristo *et al*. (2001) and Majdan & Shatz (2006)
*growth factors*		
neurotrophins	maturation of circuitry	Huang *et al*. (1999), Lodovichi *et al*. (2000) and Gianfranceschi *et al*. (2003)
IGF1	maturation of circuitry	Obata *et al*. (1999), Tropea *et al*. (2006) and Ciucci *et al*. (2007)
*GABAergic transmission*		
GABA receptors	E/I balance	Hensch *et al.* (1998), Fagiolini *et al*. (2004), Jiang *et al*. (2005) and Ponomarev *et al*. (2006)
*glutamatergic transmission*		
MGluR	activation of cellular signalling	Wang & Daw (2003), Rao & Daw (2004) and Dolen & Bear (2008)
NMDAR	activation of cellular signalling	Quinlan *et al*. (1999), Sawtell *et al*. (2003), Chen & Bear (2006) and He *et al*. (2006)
*neuromodulators*		
acetylcholine	modulator of calcium-dependent signalling	Bear & Singer (1986), Kirkwood *et al*. (1999) and Origlia *et al*. (2006)
serotonin	structural reorganization/E-I balance	Gu & Singer (1995), Kojic *et al*. (1997, 2000),
		Edagawa *et al*. (2001), Matsukawa *et al*. (2003) and
		Maya Vetencourt *et al*. (2008)
noradrenaline	modulator of calcium-dependent signalling	Bear & Singer (1986), Kirkwood *et al*. (1999) and Matsukawa *et al*. (2003)
*others*		
PirB	structural remodelling of circuits	Syken *et al*. (2006)
TNFα	synaptic scaling	Kaneko *et al*. (2008)
cannabinoid receptors	layer-specific LTD	Crozier *et al*. (2007) and Liu *et al*. (2008)
